# MotivATE: A Pretreatment Web-Based Program to Improve Attendance at UK Outpatient Services Among Adults With Eating Disorders

**DOI:** 10.2196/resprot.7440

**Published:** 2017-07-26

**Authors:** Sarah Muir, Ciarán Newell, Jess Griffiths, Kathy Walker, Holly Hooper, Sarah Thomas, Peter W Thomas, Jon Arcelus, James Day, Katherine M Appleton

**Affiliations:** ^1^ Bournemouth University Psychology Department Poole United Kingdom; ^2^ Dorset Healthcare University NHS Foundation Trust Poole United Kingdom; ^3^ i*feel Organisation Poole United Kingdom; ^4^ Patient Safety University Hospital Coventry and Warwickshire Coventry United Kingdom; ^5^ Research Centre for Behaviour Change Psychology Department Bournemouth University Poole United Kingdom; ^6^ Bournemouth University Clinical Research Unit Faculty of Health and Social Sciences Bournemouth United Kingdom; ^7^ Institute of Mental Health Nottingham University Nottingham United Kingdom

**Keywords:** Program Development, Program Evaluation, Internet, Patient Acceptance of Health Care, Assessment, Process, Feeding and Eating Disorders, Anorexia Nervosa, Bulimia Nervosa, Binge Eating Disorder

## Abstract

**Background:**

In the UK, eating disorders affect upward of 725,000 people per year, and early assessment and treatment are important for patient outcomes. Around a third of adult outpatients in the UK who are referred to specialist eating disorder services do not attend, which could be related to patient factors related to ambivalence, fear, and a lack of confidence about change. This lack of engagement has a negative impact on the quality of life of patients and has implications for service costs.

**Objective:**

To describe the development of a Web-based program (“MotivATE”) designed for delivery at the point of referral to an eating disorder service, with the aim of increasing service attendance.

**Methods:**

We used intervention mapping and a person-based approach to design the MotivATE program and conducted a needs assessment to determine the current impact of service nonattendance on patients (via a review of the qualitative evidence) and services (through a service provision survey to understand current issues in UK services). Following the needs assessment, we followed the five steps of program development outlined by Bartholomew et al (1998): (1) creating a matrix of proximal program objectives; (2) selecting theory-based intervention methods and strategies; (3) designing and organizing the program; (4) specifying adoption and implementation plans; and (5) generating program evaluation plans.

**Results:**

The needs assessment identified current nonattendance rates of 10%-32%. We defined the objective of MotivATE as increasing attendance rates at an eating disorder service and considered four key determinants of poor attendance: patient ambivalence about change, low patient self-efficacy, recognition of the need to change, and expectations about assessment. We chose aspects of motivational interviewing, self-determination theory, and the use of patient stories as the most appropriate ways to enable change. Think-aloud piloting with people with lived experience of an eating disorder resulted in positive feedback on the MotivATE program. Participants related well to the stories used. Nonetheless, because of feedback, we further modified the program in line with patients’ stage of change and addressed issues with the language used. A consultation with service staff meant that we could make clear implementation plans. Finally, a randomized controlled trial is currently underway to evaluate the MotivATE program.

**Conclusions:**

Using intervention mapping, we have developed a novel pretreatment Web-based program that is acceptable to people with eating disorders. To our knowledge, this is the first such program. The model of development described here could be a useful template for designing further programs for other difficult-to-engage populations.

## Introduction

Eating disorders (including anorexia nervosa, bulimia nervosa, other specified feeding or eating disorder, and binge eating disorder) have a prevalence rate of 1 in 600,000–725,000 people in the UK, affecting up to 6.4% of adults at any time [1,2]. These disorders are typically characterized by people over-evaluating themselves based on their weight and shape or by people engaging in eating behaviors as a mechanism for coping with difficult emotions or relationships [3,4]. Eating disorders have the highest mortality rate of all mental health conditions [5], and the number of affected individuals being diagnosed and admitted to inpatient care in the UK has been increasing by 7% each year since 2009 [2]. Early assessment and intervention are essential for patient outcomes [6,7] and the quality of life of patients and their carers [1], but it is reported that as many as 44% of people with eating disorders do not access mental health care treatment for their eating disorder [8].

After a person in the UK initially seeks help for his or her eating disorder (eg, from primary care), he or she is usually referred to a specialist eating disorder service for an initial assessment appointment. However, research highlights that up to a third of people referred for specialist psychological treatment do not access the services and that 16.4% of these people simply do not attend their first scheduled assessment appointment [9]. Reasons for this lack of engagement may include having had an eating disorder for a long time; laxative abuse; and symptoms of depression, substance abuse, or borderline personality disorder but predictors of nonattendance are still relatively unknown, as current quantitative studies report extremely low sample sizes [10]. Other barriers to psychological treatment may include stigma, poor mental health literacy, a perceived lack of a need for treatment, unhelpful past experiences with treatment, a fear of change, low motivation to change, service restrictions, and cost [11]. Aiming to address some of these barriers to psychological care, we have developed MotivATE, a Web-based, motivation enhancement-focused program that specifically seeks to improve patient engagement at the initial assessment appointment, that is, at the point that a person is referred to an eating disorder service. This paper describes the development of this program.

## Methods

We developed the MotivATE program using intervention mapping, which recognizes three phases of program development: needs assessment, program development, and evaluation [12]. As recommended by Bartholomew et al [12], our development team included developers (researchers at the university; SM, KA, KW, JD, ST, and PT), implementers (consultants working within adult eating disorder services; CN and JA), and prospective program participants (people with lived experience of having and recovering from an eating disorder; JG and HH). We also used a person-based approach to develop MotivATE to ensure that the user experience was at the heart of each stage of intervention development [13]. As highlighted in the introduction, there are a number of personal barriers to engagement with specialist services for people with eating disorders; therefore, it was essential that we had a deep understanding of their experience and needs to increase the likelihood that the intervention would be relevant, acceptable, and useful for our target group.

### Needs Assessment

The aims of the needs assessment were to understand the problem of nonattendance at adult eating disorder services and to consider both patients’ personal barriers and service-level barriers that the program would need to address. In line with the person-based approach, our first step was to review our own qualitative research with people who described themselves as not wanting to receive treatment for an eating disorder [4] and those who described themselves as in recovery [14,15], as these cases were the source of the idea for the program. Thus, we considered this work alongside other published work relating to patients’ experiences of having an eating disorder and attending treatment. To understand the impact of nonattendance at specialist eating disorder services, we also conducted a telephone-based service provision survey with four lead consultants for outpatient services in the UK. The survey questions consisted of a mix of close-ended and open-ended questions and asked about the number of referrals, the number of patients who did not attend an assessment appointment, and the processes used to try to engage patients. The survey study received university ethics approval, and each service gave verbal consent to use of its data.

### Program Development and Evaluation

Bartholomew et al [12] outline five steps of program development, and we detail these below, along with a description of the methods used to implement the steps.

#### 1. Creating a Matrix of Proximal Program Objectives

The needs assessment, team discussions, and consultations with eating disorder-focused charities enabled us to determine (a) the objectives of the program, (b) the behaviors that potential users needed to engage in, (c) the factors (or determinants) influencing these behaviors, and (d) the target population (and any subgroups) who would use the program.

#### 2. Selecting Theory-Based Intervention Methods and Practical Strategies

We reviewed the current theory related to increasing motivation to change, including the current evidence relating to eating disorders, and then considered practical strategies for implementing this theory. As per the person-based approach, this enabled us to develop guiding principles for the design of the program [13].

#### 3. Designing and Organizing a Program

The team designed the structure and content of the intervention during team meetings, and we used open-source LifeGuide software to create the MotivATE platform [16]. We started with a booklet already devised by CN and JG that aimed to help people think about the recovery process, as this was already well received by existing service users. This booklet was based on motivational activities commonly used to improve motivation to change. We adapted it to (a) address some of the barriers to engagement identified in the needs assessment stage and incorporate the language and style of a person-based approach, (b) incorporate new knowledge gained from revisiting motivational theory, (c) better address the program aims (ie, to be specifically about attending an assessment appointment, rather than about beginning therapy), and (d) enhance usability via a Web-based platform (increasing interactivity with videos, quizzes, and click-throughs) and enable tailoring based on the person’s stage of change. We also adapted the content based on our evaluation of the program (see below).

People with experience of an eating disorder then qualitatively evaluated our initial prototype of the program via think-aloud interviews to understand beliefs about the relevance, acceptability, and usability of the program [17]. We recruited participants from the university and a local eating disorder-focused charity via advertisements asking for people with experience of an eating disorder to evaluate a web program aimed at preparing people for their assessment, resulting in an opportunity sample of 12 participants (5 with binge eating disorder (4 female, 1 male), 4 with anorexia nervosa (1 male, 3 female), 2 with bulimia nervosa (both female), and 1 with an eating disorder not otherwise specified (female)). The think-aloud interviews followed an unstructured approach and asked participants to verbalize their thoughts as they occurred. Participants were asked to think back to when they were referred to an eating disorder service and to think aloud as they used the program. The researcher could then observe in-the-moment reactions to the program and used prompts (such as “What are you thinking about now?” or “Can you please explain why you chose to click on that option?”) to encourage participants’ verbalization and to understand their experience of the program. We subsequently used participant feedback to modify the program to make it more acceptable and relevant for program users.

#### 4. Specifying Adoption and Implementation Plans

This phase involved making plans for the delivery of the program: two team members demonstrated the program to staff at a local eating disorder service that was typical of outpatient services across the UK and invited feedback about implementation.

#### 5. Generating Program Evaluation Plans

The final stage of the intervention mapping described by Bartholomew et al [12] is to generate plans to evaluate the program.

## Results

### Needs Assessment

#### Literature Review

The idea to develop a program to address attendance at psychological services for eating disorders originated from previous qualitative research conducted by the authors [4,14-15]. Qualitative studies examined people who wished to maintain their eating disorder [4] and those who wished to recover [14,15] to understand their lived experience of having an eating disorder and attending treatment. The results from both types of studies identified the extreme ambivalence that people with eating disorders experience: people with eating disorders can perceive their eating disorder to be a coping mechanism, and at the same time recognise its consequence on their health, their family, and their future lives. Other qualitative research has also identified this ambivalence [18-21]. Ambivalence is a “natural phase in the process of change,” but it can cause patients to get stuck in a phase of inaction, and therefore negatively impact treatment engagement [22]. People with eating disorders also experience barriers to recovery, including low self-efficacy and (often inaccurate) preconceived expectations of what treatment could entail [4,14,20]. Leavey et al [21] conducted qualitative interviews with 13 people with eating disorders who did not attend an assessment appointment. Underlying participants’ experiences was an ambivalence about change that ultimately stopped them from attending their appointment. Participants also reported feelings of mistrust of health professionals, dissonance between their own and professional views of the disorder, fear of abandonment, comorbid mental health issues, previous negative experiences with services, misguided expectations, stigma about mental health services, and long waiting times as barriers to attendance. Although initiatives exist to improve service attendance in general health care (eg, appointment reminders or the use of opt-in systems where a person is invited to make an appointment), they can deter people who are already known to be more difficult to engage [23-25]. Given the ambivalence that many people with eating disorders experience, an intervention to improve service attendance needs to enhance autonomous motivation to change through active behavioral change techniques. Here, we chose a Web-based approach, as it would be less resource intensive for the service and more acceptable to patients who would not need to approach the service directly.

#### Service Provision Survey

Waller et al [9] reported a nonattendance rate of 16.4% in 2 South London services; we wanted to determine to what extent this was an issue in services across the UK. We sent email invitations to 9 services, and 4 (1 from the South of England, 2 from Central England, and 1 from the North) took part. Reasons for nonparticipation included a lack of time and/or a lack of useful audit data. Table 1 presents the results from the survey. Of those who were suitable for an assessment appointment, between 10% and 32% did not attend. Similar to published research [24,25], these results suggest that the use of opt-in systems to try to improve engagement may actually increase the number that do not attend.

**Table 1:** Results of the service provision survey relating to nonattendance rates and the assessment appointment process for 2013.

	Service 1	Service 2	Service 3	Service 4
Description of assessment appointment process	Contact by telephone, then send appointment letter with date and time	Send appointment letter with date and time	Opt-in process via letter. Patient to contact service within 2 weeks	Opt-in process via letter. Patient to contact service within 2 weeks
Resources provided before assessment	Map to service	Outcome measures packet	Some monitoring or dietician advice if advised by clinician	Outcome measures packet, information about service, map to service, questions about demographics
Average length of wait (referral to assessment)	14 days	2.5 months	Not reported	34 days
n suitable for outpatient assessment (in the year 2013)	172	135	153	352
n did not opt in^a^ (% of suitable referrals)	N/A	N/A	24 (16)	86 (24)
n suitable outpatient referrals who did not attend appointment (% of suitable referrals)	33 (19)	14 (10)	25 (16)	16 (5)
Total who did not attend first assessment appointment (%)	33 (19)	14 (10)	49 (32)	102 (29)

^a^The number that did not schedule an appointment when invited to

### Program Development and Evaluation

#### Creating a Matrix of Proximal Program Objectives

The main objective of the program is to “increase attendance at an eating disorder service”. In contrast to the opt-in process detailed above, which simply asks patients to make an appointment at a convenient time, the MotivATE approach aims to increase attendance rates by fostering personal intrinsic motivation and increased self-efficacy to attend while addressing negative beliefs and expectations about the service. The target population for the program was all adults who have been referred to an outpatient eating disorder service for an assessment appointment. People in the UK are usually referred to an eating disorder service by their general practitioner, although there are other routes to treatment, depending on the structure of the local service model. Some services allow referral by any health or social care professional, and others welcome the person to refer himself or herself. Clinicians at the service review the referral letter to determine the next steps, guided by the urgency of referral, risks, etc. When the person is suitable for outpatient treatment (eg, there are no immediate risks requiring a higher level of care), the service will contact the person to invite him or her for an assessment appointment (eg, to assess eating disorder behaviors and symptoms and the need and motivations for treatment and to collaboratively develop a treatment plan). The wait times for the assessment appointment are different across the UK, ranging from 4 weeks to 6 months. It is when inviting the person for assessment that we intend to include the invitation to use MotivATE.

People who have been referred to the service may be in one of three of the five stages of change [26]. First, patients may be in the precontemplation stage of change; that is, they may not believe that they have an eating disorder and are not intending to change. Second, many are likely to be in the contemplation stage; they may be aware of the pros and cons of change but highly ambivalent about doing so. Third, those in the preparation stage may have plans to change within the next month but may not have high enough confidence (or self-efficacy) to do so. The last two stages of change include the action stage (those who have been making changes in the last 6 months) and the maintenance stage (those who have made a change and are working to prevent relapse). People in these two stages would not be target users of the program, as they are likely to be already engaging with a treatment program. Table 2 provides details of how theses stages of change could impact program requirements.

**Table 2:** Subgroups of potential program users.

Subgroup	Distinction	Implications for performance objectives	Example of performance objectives
Precontemplation	Not considering changing eating disorder and/or may not believe they have an eating disorder	Least likely to attend an assessment; require education about eating disorders and stories from others to start recognizing own problematic behaviors	Starting to recognize eating disorder experience and becoming educated about the cons of the disorder
Contemplation	Extremely ambivalent group; may be swaying between attending and not attending	Ambivalence is a salient determinant	Weighing pros and cons of change and addressing ambivalence
Preparation	Accept a need for change but may not have high enough confidence to do so	Low self-efficacy is an important determinant	Feel more confident about ability to change, think about assessment and what to expect, and prepare to attend

**Table 3:** Program objectives for MotivATE.

Performance objective	Determinants			
	Ambivalence about change	Self-efficacy	Recognition of need to change	Expectations about assessment
Attend assessment appointment	Recognize ambivalence but attend to learn more	Feel confident and in control of assessment appointment	Recognize possible need to change and attend to learn more	Have realistic expectations of what is involved at the assessment appointment

The needs assessment and the identification of the target group enabled us to ascertain four main determinants that the program needed to address: (1) patient ambivalence about change, (2) low patient self-efficacy, (3) recognition of the need to change, and (4) expectations about assessment. This resulted in the final matrix of proximal program objectives outlined in Table 3.

#### Selecting Theory-Based Intervention Methods and Practical Strategies

The main objective of the program is to increase motivation to attend an assessment appointment. Motivational therapies are advocated in the treatment of eating disorders [27] and are effective at improving motivation to change [28-30]. These interventions are based on or closely adapted from the principles of motivational interviewing [22,31,32] and involve addressing and working through ambivalence and initiating “change talk” from patients, encouraging them to make choices that fit with their own goals and values. Specifically, psychoeducation, examining the pros and cons of symptoms, experimental strategies, and exploring personal values are four methods proposed to help ambivalent eating disorder patients move through the stages of change [33]. A service traditionally delivers these methods face to face once a person has engaged with the service, but there is growing evidence for Web-based delivery [34,35] and for offering intervention prior to engagement among those with eating disorders [36].

We also drew on self-determination theory (SDT) for the intervention, which is in keeping with the person-based approach. SDT recognizes that human behavior centers on three innate needs: autonomy, competence, and relatedness [37]. People need to feel in control and autonomous to be able to internalize behavioral change and perceive it as important to their own values and goals. Choice is particularly important to patients with eating disorders, who may fear that control will be taken away from them during treatment [18,38]. It may also have a direct impact on service engagement. Indeed, when people with eating disorders referred for inpatient treatment had full choice (even including whether they would stay at all), they were less likely to drop out of treatment [39]. Competence refers to the person’s self-efficacy or confidence to perform the behavioral change. Relatedness refers to the person’s ability to relate to others and foster supportive relationships. As in motivational interviewing, the clinician’s role, per SDT, is to provide supportive autonomy by encouraging patients to take the lead in making a change and to help them to internalize recognition of change as an important goal for themselves [40]. In MotivATE, we relied on video and written accounts of people’s experiences and success stories of attending an assessment appointment (also known as narrative communication [41]) to enhance a sense of relatedness and to address the four determinants of the program (increase recognition of the need to change, increase self-efficacy, address expectations, and address ambivalence). For example, people could recognize their own situation in the stories (increase recognition), and reading success stories from others can increase self-efficacy and the intention to change [42].

The program objectives highlighted in the previous stage and the examination of theory and practical strategies allowed us to develop guiding principles, outlined by the person-based approach, for the program (Table 4).

**Table 4:** Guiding principles for MotivATE.

Intervention design objectives	Key feature(s)
To be delivered before any formal contact with the service and to address expectations about assessment (ie, address the question of “What will they *do* to me?”)	Provide a digital intervention with education about service and assessment through interactive quizzes and stories about others’ experiences
To address and acknowledge ambivalence and to enhance or maintain motivation to attend	Build autonomous motivation, address patients’ mixed feelings about change and link change to their own personal goals and values, tailor program to stages of change, provide psychoeducation about eating disorders, and highlight choice that person can make during program and when they attend assessment
To increase self-efficacy and to help patients to make their own decisions	Develop intervention user’s competence through user stories

**Table 5:** The content of the MotivATE program.

Module	Aim	Content
1. What happens at the first appointment?	Address expectations about the assessment appointment	Provides an interactive quiz to explore common misconceptions about assessment, information about the assessment appointment, and stories and videos about others’ experiences.
2. How motivated are you?	Introduce the idea of change	Introduces people to the stages-of-change model with stories of others’ experiences. Person can choose his or her stage of change.
3. Arming yourself with information	Help people to recognize problematic behaviors (precontemplation) and address ambivalence	Provides information about the pros and cons of eating disorders. Those who have selected the contemplation or preparation stage of change can complete their own tables of pros and cons and complete exercises designed to address ambivalence.
4. Preparing for your assessment	Improve confidence to attend	Includes a video of a clinician welcoming people to the assessment, and users can make plans to attend their appointment.

#### Designing and Organizing a Program

**Figure 1:**
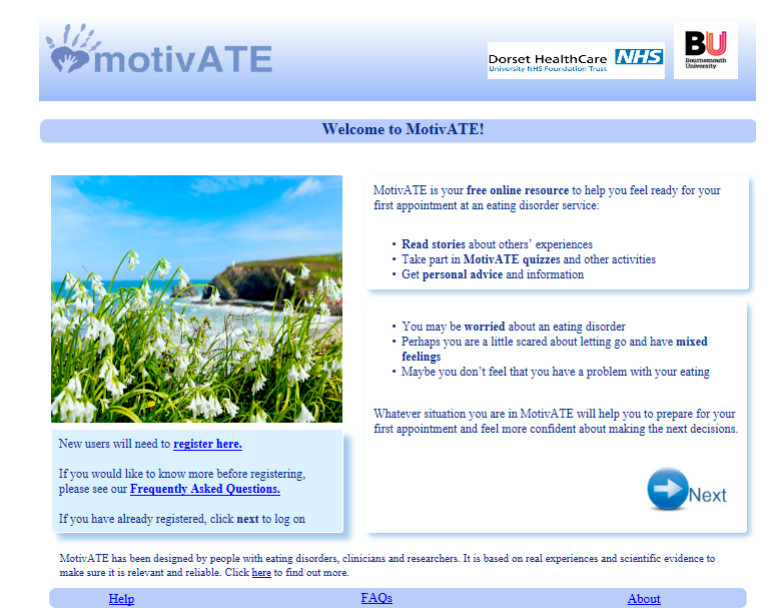
Home page of the MotivATE website.

We aimed to present a professional-looking, gender- and age-neutral program. The color scheme is blue and white, and images are based on nature, with the intention of depicting positive well-being (see Figure 1). No images of people or food are included, as we do not want the program to negatively influence people or to cause them to compare their body shape or eating behaviors to those of others. Talking-head videos of real people are included throughout the intervention, depicting men and women with different eating disorders, under the assumption that their stories will resonate with program users. The university and service logos are included on each page to convey professionalism and credibility, and it is clear from the start that people with eating disorders, researchers, and clinicians designed the intervention.

The final MotivATE program consists of four 15-minute Web-based modules (see Table 5 for details about the content). The modules are brief to accommodate program users’ potentially limited concentration levels. We wanted to design the program to give users as much choice as possible. Program users can work through the modules in any order (though a specific order is recommended) and can complete the modules all at once or as desired and when they had time. The choice of language reflects the person-based approach (see [12]), and an autonomy-supportive tone is used (eg, “you may find...” and “people have told us they feel worried about their assessment”). It is nonprescriptive and does not assume users’ experiences but does acknowledge users’ ambivalence (eg, “perhaps you are a little worried about letting go and have mixed feelings”).

People with experience of an eating disorder evaluated early versions of MotivATE. The results of the evaluations fit into four overarching themes.

##### Theme 1: Positive Perspectives on MotivATE

General comments about the MotivATE website were positive. Participants liked the esthetics of the site, describing it as “bright”, “cheerful”, and “calming”. They liked the blue color scheme and neutral images.

It’s quite a neutral image as well. I don’t think it’d be very nice if there was like images of people or anything like that so I quite like it...I think they’re right, neutral pictures, that’s always a great idea.

Kate, binge eating disorder

Participants consistently referred to the information and stories on the site as “positive” and “reassuring”. They felt that the information and stories were relevant to them; they related well to the people in the stories, and this seemed to improve self-efficacy and helped them to recognize areas for change.

That’s very good, too, as you need proof. You desperately need to see someone or hear of someone who really has got better, and these are all things that are building confidence, aren’t they?

Lily, anorexia nervosa

##### Theme 2: The Need to Tailor the Program to Different Motivational Needs

Some participants worried that the information could be inappropriate or unhelpful for those in the precontemplation stage of change. Sam, who did not believe “there was anything wrong” at the time of his assessment, gave good insight into how he would have felt at the time of referral:

After [I was] diagnosed and before [I was] assessed, not sure I would have felt any of these things...But as I was through my recovery, then yes, I probably said all of these things....

Sam, anorexia nervosa

Sam described how reading stories of others’ experiences helped him to recognize similar thinking patterns and behaviors in himself. Once he started to realize that he had an eating disorder, he sought as much information as he could. These insights helped us to tailor the stories better, and we added more psychoeducation for those in the precontemplation stage of change.

##### Theme 3: Addressing Fears About Recovery

Thinking further ahead than the assessment appointment could be “daunting and scary” (Evie, Bulimia Nervosa), and it was clear from talking to participants that the website should not be overtly about “recovery”; instead, it should “sow the seed” (Sam) and focus on how to prepare for the assessment appointment. Early versions of the program also discussed the concepts of recovery and psychological treatment, but based on the following feedback, the final program only considered the first step of the assessment appointment:

I freaked a little bit then. Thinking about change in the future. I’m going to move onto something else (chooses quiz to distract from message on page) because that’s what I would do.

Sally, eating disorder not otherwise specified

##### Theme 4: Eating Disorder-Specific Aspects of Designing a Web-Based Program

Some participants (particularly those with a history of anorexia nervosa) believed that some modules were too long, and we therefore decided to shorten the modules to no longer than 10 minutes, focusing on essential information. Participants also commented on the terminology. For example, we referred to question *scales* and motivational *exercises*, but these have connotations related to body weight, so we removed these references.

We used the results from the think-aloud interviews to modify the program, and further consultations with participants as well as consultations with charity directors and others with experience of eating disorders confirmed that the program was acceptable to users.

#### Specifying Adoption and Implementation Plans

We demonstrated the MotivATE program to clinical and administrative staff at one eating disorder service before we asked for feedback about implementing MotivATE in their practice. Two key points arose: (1) it should not impact existing service resources and staff time, and (2) the modules needed to be short enough to optimize engagement of patients. MotivATE would be a free, stand-alone program that would not require training for service staff or patients. Invitation to MotivATE would occur via the same materials as the standard invitation to attend an assessment and would therefore not impact staff time and resources. We are planning a process evaluation with program users and service staff to further explore implementation.

#### Evaluation

We have already evaluated the acceptability and usability of the intervention as part of the program development described here. A randomized controlled trial (RCT) is also currently underway to evaluate the impact of the MotivATE program on improving attendance at an eating disorder service (Trial Registration: NCT02777944).

## Discussion

This paper described the development of a pretreatment Web-based program designed to support adults with eating disorders as they prepare for an assessment appointment at a specialist outpatient eating disorder service. We have yet to establish the program’s value for and impact on service attendance rates, although the RCT will enable us to determine this. Nonetheless, we believe the program will improve attendance at assessment appointments based on comments from service users and service staff.

The combined approach of using intervention mapping and a person-based approach worked well, as it allowed us to structure our development process in a logical manner by identifying areas of need, setting out key objectives from the outset, and highlighting key theory and strategies that could be used while always ensuring that each of these components would work for the target user. Our person-based approach is a key strength of the development process. Specifically, this program needs to be acceptable, motivating, and empowering for users who already find it difficult to engage with services. Interviews with service users and service staff also revealed important shortcomings of the first version of the intervention, developed based on existing literature and theory, which we have been able to address and tailor more to service user needs (ie, shorter sessions, more psychoeducation for those in an earlier stage of change, and more focus on assessment rather than the full recovery process). The use of existing qualitative literature and theory was also fundamental to our intervention design since they not only ensured an evidence-based approach to program development, but they also enabled the inclusion of a variety of processes designed to increase motivation, and allowed us to consider the characteristics of populations that can be difficult to engage. Our needs assessment has also demonstrated the potential usefulness of the MotivATE program across services in the UK, although one weakness of our service provision survey is that for practical reasons (resources and time demands making it difficult for services to engage), we included only four services. Nonetheless, the study still provides a more generalized view than existing studies, which have focused only on one area of the UK [9].

One difficulty in the design phase was the production of a Web-based intervention that utilized the principles of motivational interviewing and SDT, which rely heavily on face-to-face interaction. Use of a person-based language style, which enabled us to provide autonomy support [13], and optional interactive tasks, which allowed people to engage in motivation-to-change techniques, overcame this difficulty. Again, by working with participants to qualitatively evaluate the modules, we could adapt the language to ensure that it was motivating for them. Nonetheless, a weakness of the program is that we have not yet assessed whether the intervention improves motivation to change in individuals, and this is a plan for a future study.

Despite positive feedback regarding the acceptability of the MotivATE program, a key unknown is whether people will register for and engage with it, particularly as Web-based interventions are known to have low rates of engagement [43]. Our RCT will give us some details about this, but further research is required to understand uptake of and engagement with Web-based interventions, particularly in populations already known to be difficult to engage by face-to-face intervention. It seems that consideration of user engagement should form part of the intervention design [44] and could be implemented within the intervention mapping process. A developing area of interest regarding uptake is the study of how peripheral cues, such as esthetic appeal, may improve website stickiness [45]; however, this concept is yet to be applied to the design of Web-based health behavioral change interventions, and further research is needed.

To our knowledge, the MotivATE program is the first intervention designed for delivery to eating disorder patients prior to formal contact with an eating disorder service with the aim of enhancing engagement with face-to-face services. If the program is effective, it could act as a template for the design of similar pretreatment Web-based programs for other patient groups in which ambivalent attitudes and/or a lack of engagement with services may be an issue.

## Abbreviations

RCT: randomized controlled trial
SDT: self-determination theory
